# A Comprehensive Review of Fluid Resuscitation Strategies in Traumatic Brain Injury

**DOI:** 10.3390/jcm14176289

**Published:** 2025-09-05

**Authors:** Mairi Ziaka, Wolf Hautz, Aristomenis Exadaktylos

**Affiliations:** 1 Department of Emergency Medicine, Inselspital, University Hospital, University of Bern, 3012 Bern, Switzerland; wolf.hautz@insel.ch; 2 Academic Department of Emergency Medicine, School of Medicine, University of Cyprus, 1678 Nicosia, Cyprus; docaris@protonmail.com

**Keywords:** arterial blood pressure, blood transfusion, cerebral perfusion pressure, fluids, fluid management, hemodynamic monitoring, multimodal monitoring, traumatic brain injury

## Abstract

The current management of severe traumatic brain injury (TBI) focuses on maintaining cerebral perfusion pressure (CPP) to prevent or minimize secondary brain injury, limit cerebral edema, optimize oxygen delivery to the brain, and reduce primary neuronal damage by addressing contributing risk factors such as hypotension and hypoxia. Hypotension and cardiac dysfunction are common in patients with severe TBI, often requiring treatment with intravenous fluids and vasopressors. The primary categories of resuscitation fluids include crystalloids, colloids (such as albumin), and blood products. Fluid osmolarity is a critical consideration in TBI patients, as hypotonic fluids, such as balanced crystalloids, may increase the risk of cerebral edema development and worsening. Hyperosmolar therapy is a common therapeutic approach in patients with intracranial hypertension; however, its use as a resuscitation fluid is not associated with benefits in patients with TBI and is not recommended. Given the contradictory results of trials on blood transfusion strategies in patients with TBI, the transfusion approach should be tailored to individual systemic and cerebral physiological parameters. The evaluation of recent randomized clinical trials will provide insight into whether a liberal or restrictive transfusion strategy is preferred for this patient population. Hemodynamic and multimodal neurological monitoring to assess cerebral oxygenation, autoregulation, and metabolism are essential tools for detecting early hemodynamic alterations and cerebral injury, guiding resuscitation management, and contributing to improved outcomes.

## 1. Introduction

TBI is a major public health problem with estimated annual cases reaching sixty-nine million individuals globally [1]. The pathophysiology of TBI is multifactorial, involving an initial primary injury directly associated with external mechanical forces and secondary injuries related to cellular, biochemical, and inflammatory cascades triggered by the primary insult [2,3]. Cornerstones of TBI management predominantly focus on monitoring intracranial pressure (ICP) and maintaining CPP to avoid or minimize secondary brain injury, limit cerebral edema, and diminish primary neuronal damage by addressing risk factors that contribute to it, such as hypotension and hypoxia [4,5,6,7]. Hypotension is common in patients with TBI, with underlying multifactorial pathophysiologic mechanisms often associated with concomitant hemorrhagic complications from various traumatic injuries [8]. Moreover, cardiac dysfunction secondary to sympathetic activation and a catecholamine storm following TBI is assumed to play a pivotal role in the development of hypotension associated with ventricular dysfunction and vasoplegia, especially in patients with severe TBI [9,10,11,12]. Moreover, experimental research demonstrates that isolated severe TBI leads to an immediate and significant decrease in plasma volume, accompanied by an elevation in hematocrit and a decrease in pH, likely associated with vascular leakage [13].

Clinical studies highlight that systolic blood pressure (SBP) values below 90 mmHg are associated with worse outcomes, poor neurological prognosis, higher mortality, and longer intensive care unit (ICU) stays [14,15,16], emphasizing the importance of preventing and rapidly treating hypotension. Notably, the goal of resuscitation should be the maintenance of normotensive blood pressure levels, as both hypotension and hypertension can have harmful effects. To achieve euvolemia, careful fluid administration is commonly the initial approach, while vasoactive agents are used for patients who do not respond to fluid therapy [17]. According to the consensus committee at European Society of Intensive Care Medicine (ESICM) LIVES 2016 (October 2016), crystalloids should be used as first-line resuscitation fluids in neurocritically ill patients with low blood pressure. Fluid administration should be guided by multiple hemodynamic parameters, such as arterial blood pressure and fluid balance, to improve fluid therapy, while additional variables—including cardiac output (CO), mixed venous oxygen saturation (SvO_2_) and central venous oxygen saturation (ScvO_2_), blood lactate, and urinary output—may further optimize fluid management [18]. Nevertheless, it is well established that crystalloids distribute within a small expandable space, and large volumes must frequently be administered to restore sufficient blood pressure [4,19], which may lead to hypervolemia, a condition associated with poor prognosis [20]. Moreover, this approach may lead to elevated hydrostatic pressure in damaged vessels, induce dilutional coagulopathy, and promote the formation of hemostatic blood clots [21,22,23,24], while also contributing to hypothermia [25,26]. On the other hand, colloidal solutions have the property of persisting intravascularly for longer periods, mainly due to their high molecular weight and the resulting difficulty in crossing the vascular endothelium [27,28]. Data regarding the use of colloids in patients with TBI are scarce. A retrospective cohort study of patients with severe TBI found no association between pentastarch administration and mortality [29]. However, the SAFE-TBI study highlighted that, compared to saline, the administration of albumin in TBI patients is associated with significantly higher mortality [30]. In addition, in clinical studies including patients with subarachnoid hemorrhage (SAH), the use of colloids was associated with worse 6-month Glasgow Outcome Scores [31,32].

Anemia is very common among critically ill patients, affecting nearly 75% of ICU patients and being associated with adverse outcomes [33,34]. Nonetheless, transfusion of blood products is linked to several complications, such as transfusion-associated lung injury, hypervolemia, secondary infections, and immunosuppression [35,36]. While a restrictive transfusion strategy is recommended over a liberal strategy for most patient populations in the general ICU, the optimal transfusion strategy in patients with TBI remains less clear [37,38,39]. Indeed, given the low certainty of the existing data, the European Society of Intensive Care Medicine was unable to make a recommendation regarding hemoglobin targets and transfusion strategy (liberal vs. restrictive) in its recent clinical practice guidelines for neurocritically ill patients [37].

In the present work, we aim to comprehensively summarize the existing evidence on fluid resuscitation therapy in critically ill adult patients with TBI, with an emphasis on pathophysiological issues such as the role of blood–brain barrier (BBB) disruption, alterations in cerebral autoregulation, and the impact of systemic hemodynamics. Moreover, we focus on the goals of fluid resuscitation, hemodynamic monitoring, and multimodal neuromonitoring, types of fluids, and transfusion strategies.

## 2. Methods

A comprehensive, non-systematic literature search was conducted using PubMed to identify relevant studies focusing on fluid management in patients with TBI. The search terms included “*traumatic brain injury*”, “*acute brain injury*”, “*arterial blood pressure*”, “*cerebral perfusion pressure*”, “*intracranial pressure*”, “*fluid management*”, “*fluid resuscitation*”, “*early goal-directed therapy*”, “*fluid responsiveness*”, “*hemodynamic monitoring*”, “*multimodal monitoring*”, “*crystalloids*”, “*balanced crystalloids*”, “*colloids*”, “*albumin*”, “*hypertonic saline*”, “*blood transfusion*” and “*critical care*”. Boolean operators (AND, OR) and truncations were applied to refine and optimize search results. The search concentrated on articles published in English over the past 20 years (2005–2025) in order to capture contemporary evidence, as advances in multimodal monitoring, fluid resuscitation strategies, transfusion practices, and overall neurocritical care management have evolved substantially over the past two decades; while important foundational works published before this period were included to provide historical context and a solid basis of understanding. Manual screening of references from selected studies was carried out to identify additional relevant literature.

### 2.1. Pathophysiological Issues

#### 2.1.1. Blood–Brain Barrier Disruption

**Figure 1:**
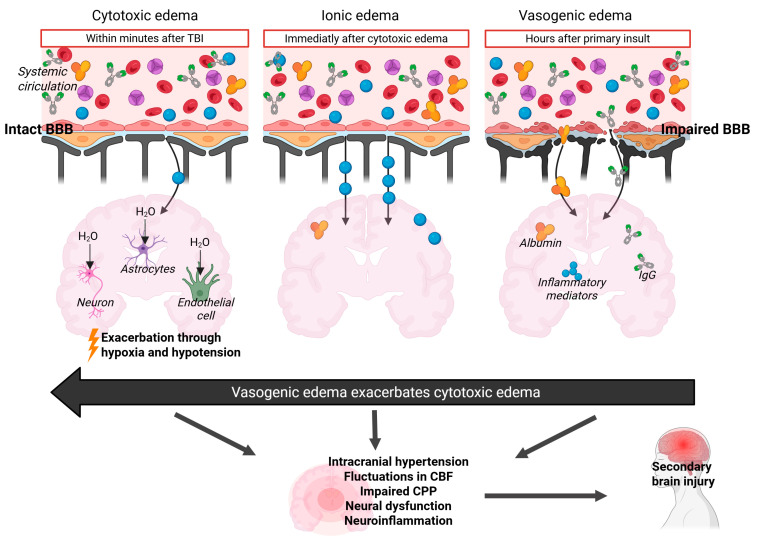
Pathophysiology of cerebral edema in patients with TBI. BBB: blood–brain barrier; CBF: cerebral brain flow; CPP: cerebral perfusion pressure; H_2_O: water.

Subsequent to the primary trauma, BBB disruption occurs within hours of the initial traumatic insult and can persist for extended periods, even years, contributing to tissue injury, cerebral edema, neural dysfunction, and inflammatory reactions [40,41,42,43]. BBB breakdown and the resulting hyperpermeability lead to the extravasation of fluid and plasma-derived factors into the perivascular space, along with the accumulation of immune cells. This elevates extracellular oncotic pressure, causing fluctuations in cerebral blood flow (CBF), increased ICP, and cerebral edema—a pathophysiological phenomenon known as vasogenic edema (Figure 1) [44,45,46]. Another consequence of TBI is cellular (cytotoxic) edema, which arises from ion channel activation and/or ionic pump failure, driving water influx from the interstitial space into intracellular compartments of astrocytes, endothelial cells, and neurons, thereby contributing to BBB impairment [44,47,48]. Importantly, the breakdown of the BBB can be further exacerbated by secondary factors such as edema, disrupted brain activity, microglial activation, astrogliosis, and neuroinflammation, all of which contribute to its continued disruption (Figure 1) [49,50].

It is well established that the BBB has notable passive permeability to free water, in contrast to its restricted permeability to electrolytes and other solutes [51,52]. Nevertheless, an acute decrease in plasma osmolality, as seen in water intoxication and hypotonic hyponatremia, creates an osmotic gradient that drives water into the brain compartment, leading to increased intracranial water content and brain edema [53,54,55].

Under normal conditions, one of the brain’s immediate protective mechanisms against hypotonic states is the hydrostatic water shift from the brain parenchyma to the cerebrospinal fluid, and subsequently into the systemic circulation [56,57,58]. However, one might argue that in patients with a disrupted BBB, such as those with TBI, this protective mechanism is impaired, potentially exacerbating cerebral edema and increasing ICP. Furthermore, neuronal cells adapt to the increased intracerebral water volume through volume regulatory decrease, a pathophysiological compensation mechanism involving the active transport of osmotic solutes and electrolytes, such as sodium, potassium, and chloride, from the intracellular to the extracellular space. Additionally, BBB endothelial cells and other cellular components of the neurovascular unit contribute to this process by secreting water into the intravascular compartment [59,60,61,62]. Nonetheless, in brain-injured patients, BBB breakdown compromises its ability to regulate the homeostasis of water, osmotic solutes, and electrolytes, making hydrostatic pressure differences the primary driver of water shifts between the intra- and extravascular spaces [62].

It is assumed that BBB disruption is common in patients with TBI, particularly those with severe TBI, and may significantly impact the effectiveness of fluid management and long-term outcomes. Clinical studies in TBI patients highlight the leakage of large-molecular-weight proteins, such as albumin and immunoglobulin G (IgG), due to BBB breakdown. This leads to elevated concentrations of these proteins in the brain, which has been associated with increased ICP, suggesting that exogenous administration of albumin may exacerbate cerebral edema and ICP [63]. Indeed, albumin is a large molecule that, due to BBB disruption, may translocate from the intravascular space into the brain parenchyma, where its osmotic properties pull water into the tissue, possibly worsening cerebral edema (Figure 1) [64].

#### 2.1.2. Cerebral Autoregulation

**Figure 2:**
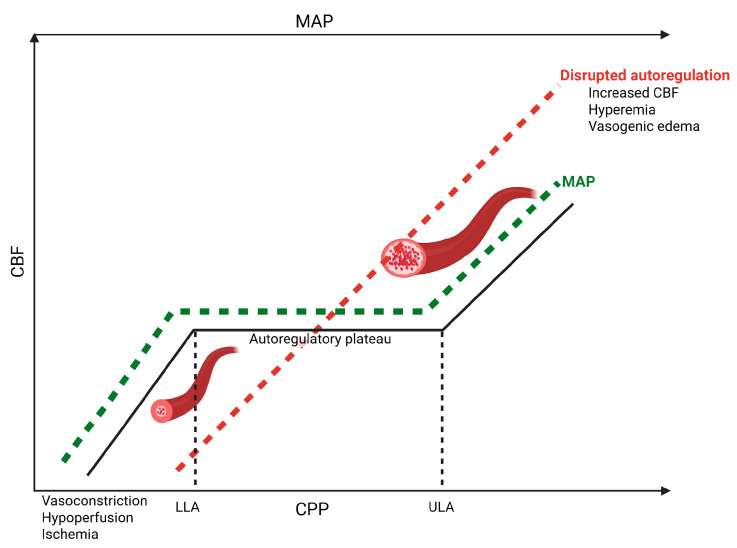
When cerebral autoregulation is intact, reactive changes in vascular resistance and corresponding adjustments in cerebral vessel diameter maintain CBF despite fluctuations in arterial blood pressure. This mechanism prevents both ischemia and hyperemia by preserving adequate perfusion, up to the upper and lower limits of autoregulation, thus meeting the brain’s metabolic demands. In patients with TBI, these autoregulatory mechanisms are often impaired, leading to secondary hypo- and/or hyperperfusion. Both conditions are harmful, potentially resulting in ischemia and hyperemia, respectively, and contribute to secondary brain injury. Autoregulation-guided management of blood pressure, ICP control, and individualized CPP targets—alongside optimal fluid therapy, the use of vasopressors, and advanced hemodynamic and multimodal neuromonitoring—may reduce the risk of secondary complications and improve functional outcomes and patient prognosis. CBF: cerebral blood flow; CPP: cerebral perfusion pressure; ICP: intracranial pressure; LLA: lower limit of autoregulation; MAP: mean arterial pressure; TBI: traumatic brain injury; ULA: upper limit of autoregulation.

Cerebral autoregulation is a protective homeostatic mechanism aimed at maintaining consistent CBF across a wide range of arterial blood pressures to prevent ischemia and hyperemia, ensuring the brain’s metabolic demands are met [65,66]. Since CPP is derived from mean arterial pressure (MAP) minus ICP, CBF remains stable through reduced cerebrovascular resistance or vasodilation in response to decreases in MAP or CPP [67]. The underlying physiologic mechanisms of CBF autoregulation are not fully understood; however, key mechanisms are postulated to include genetic, myogenic, metabolic, endothelial, and neurogenic processes (Figure 2) [68]. The myogenic mechanism of cerebral autoregulation depends on the ability of cerebral arteries to change diameter in response to alterations in blood pressure, transmural pressure, and blood flow, leading to dilation and constriction to maintain stable CBF [69,70,71,72,73]. Extensive research indicates that, among pial arterioles, larger cerebral vessels play a significant role in CBF autoregulation [74]. Changes in the diameter of these larger vessels in response to perfusion pressure alterations are related to their innervation, with constriction occurring due to adrenergic stimulation and dilation following cholinergic activation [75,76,77]. Mechanoreceptors in the endothelial cells of cerebral blood vessels are sensitive to changes in transmural pressure and shear stress, leading to the secretion of mediators such as nitric oxide, endothelin, prostacyclin, and 20-hydroxyeicosatetraenoic acid, which regulate the tone of vascular smooth muscle cells. Nitric oxide and prostacyclin cause vasodilation, increasing cerebral blood volume, while endothelin-1 and 20-hydroxyeicosatetraenoic acid lead to vasoconstriction, producing the opposite effect [68,71,73]. Additional modulators of CBF include partial pressure of carbon dioxide (pCO_2_) and partial pressure of oxygen (pO_2_) levels, with arterial hypoxia and hypercapnia leading to increased CBF through vasodilation to maintain normal brain oxygenation. Conversely, reductions in CBF are associated with arterial hyperoxia, also aiming to preserve normal brain tissue oxygenation [78,79,80,81,82].

The physiological goal of cerebral autoregulation is to maintain CBF within a specific range despite fluctuations in CPP. Under normal conditions, a low CPP leads to increased cerebral blood volume and ICP due to compensatory vasodilation. Conversely, elevated CPP results in reduced cerebral blood volume through compensatory vasoconstriction [83,84,85]. Lassen (1959) described cerebral autoregulation as a triphasic curve, characterized by lower and upper limits with a relative plateau between them (Figure 2) [65]. Outside the range of lower and upper limits of autoregulation (LLA and ULA), the pressure-flow relationship becomes more passive, and CBF can no longer be adequately regulated, potentially resulting in either hypoperfusion and hypoxic injury or hyperemia, cerebral edema, and BBB disruption, depending on the direction of the deviation [86,87]. It is well established that cerebral autoregulation is significantly impaired in neurocritically ill patients with TBI. Interestingly, this impairment can occur even when CPP and CBF values are within normal ranges [88,89,90].

Cerebral autoregulation can be assessed at the bedside by an experienced ICU physician through a MAP challenge—typically involving a 10% increase in MAP (approximately 10–15 mmHg) for up to 20 min—in euvolemic patients with an intact cranium, achieved via fluid administration and vasoactive agents. In patients with intact autoregulation, this MAP elevation leads to a reduction in cerebral blood volume and ICP through compensatory vasoconstriction. It has been suggested that in such patients—particularly those demonstrating a decrease in ICP—higher CPP targets may offer therapeutic benefit. In contrast, performing a MAP challenge in patients with impaired cerebral autoregulation is associated with a significant passive increase in ICP [91]. In addition to the MAP challenge, various techniques have been investigated to assess cerebral autoregulation. Radiologic methods include computed tomography (CT), positron emission tomography (PET), and magnetic resonance imaging (MRI). Intermittent monitoring can be performed using transcranial Doppler (TCD) ultrasound, which measures cerebral blood flow velocity in the anterior circulation and allows calculation of indices such as the mean velocity index (Mx). For continuous monitoring, the cerebrovascular pressure reactivity index (PRx)—which quantifies the correlation between slow waves of MAP and ICP—is commonly used. Near-infrared spectroscopy (NIRS) offers a non-invasive approach to assess regional cerebral oxygenation as a surrogate for CBF [92]. However, all of these modalities have inherent limitations related to accuracy and patient-specific factors. Among them, the PRx is the most extensively studied tool for assessing cerebral autoregulation through vasoreactivity [91].

#### 2.1.3. Arterial Blood Pressure and Central Venous Pressure

Perfusion pressure is dependent on upstream (e.g., arterial) and downstream (e.g., venous) pressures, with elevated venous pressures and decreased arterial pressures leading to lower perfusion pressures [93,94]. Experimental research in animal models of TBI has highlighted that elevations in central venous pressure (CVP) are associated with higher ICPs, intracranial hypertension, and cerebral edema due to the limitation of cerebral venous outflow [95,96]. Moreover, other factors can influence cerebral venous outflow, such as the application of positive-end expiratory pressure (PEEP), which may elevate mean intrathoracic pressure, increasing ICP due to impaired venous drainage. Furthermore, increased intrathoracic pressure can potentially decrease MAP, leading to subsequent disruption of CBF [97]. Stepwise PEEP elevation increases CVP, with a 12 cmH_2_O rise in PEEP causing a more than 4 mmHg increase in CVP [98,99]. Indeed, it has been demonstrated that an increase in PEEP by one centimeter water (H_2_O) leads to an ICP elevation of 0.31 mmHg by restricting intracranial venous return [100]. Moreover, the impact of PEEP on ICP is influenced by multiple factors, including respiratory mechanics, chest wall elastance, lung recruitability, baseline ICP, and intracranial compliance [101,102,103,104]. As a result, an increase in venous pressure can be transmitted back to the intracranial compartment when ICP is lower than either CVP or PEEP in mechanically ventilated patients with brain trauma. Brain compliance can also be compromised when several adverse factors act together, including hypotonic fluid administration, increased CVP, or recent brain injury with associated edema [94].

## 3. Fluid Management in Patients with TBI

### 3.1. Goals of Fluid Resuscitation

#### 3.1.1. Arterial Blood Pressure

The fundamental goal of fluid management in patients with TBI is to optimize cerebral perfusion, oxygenation, and substrate delivery to the brain while minimizing secondary brain injury [94,105]. Moreover, given that profound elevations in CPP may be associated with perilesional edema, it is especially important in patients with preserved cerebrovascular autoregulation to prevent excessive CPP fluctuations, as they can lead to both vasoconstriction and vasodilation [106].

As mentioned above, it has been convincingly demonstrated that systolic hypotension (e.g., SBP below 90 mmHg) is associated with worse outcomes [107]. However, accumulating research highlights that even an SBP below 110 mmHg may negatively impact mortality. A recent retrospective study of 154,725 patients with TBI demonstrated that the risk of in-hospital mortality decreased as SBP increased, plateauing at 110 mmHg across all age groups, including patients aged 50–69 years [108]. A retrospective study involving patients with severe TBI proposed redefining hypotension and optimizing hemodynamic goals for this population. The study suggested that the threshold for hypotension should be adjusted based on age. Specifically, patients aged 60 years or younger should be considered hypotensive at a systolic SBP of less than 100 mm Hg, while for older patients, hypotension should be diagnosed at an SBP of less than 120 mm Hg [109]. A systematic review and meta-analysis involving over 380,000 patients with moderate to severe TBI found that hypotension, defined as a SBP below 90 mm Hg, significantly impacts mortality, highlighting the need for prompt management to maintain blood pressure within recommended thresholds [8].

In patients with severe TBI, the European guideline on the management of major bleeding and coagulopathy following trauma recommends targeting a MAP of ≥80 mmHg [110]. However, the Brain Trauma Foundation (BTF) and the latest French guidelines for the management of patients with severe TBI in the first 24 h recommend a different approach, particularly maintaining SBP above 100 mmHg for patients aged 50 to 69 years and at ≥110 mmHg for patients aged 15 to 49 or over 70 years [111,112]. Nonetheless, the upper permissible limit of SBP is not clearly defined, nor are the recommendations for the management of arterial hypertension [113].

As mentioned above, TBI results in immediate and excessive catecholamine surges, which, among other effects, may cause arterial hypertension that, despite its protective role in maintaining CPP, is associated with poor outcomes [114,115,116]. It is assumed that the direct transmission of elevated SBP to the cerebral capillaries increases hydrostatic pressure at the capillary level, worsening cerebral edema—a process that is more pronounced due to impaired autoregulatory compensatory mechanisms, particularly at the site of injury [113,117,118].

#### 3.1.2. Volume Status

It has been demonstrated that fluid overload has a detrimental impact on patients with acute respiratory distress syndrome (ARDS) and sepsis, leading to worse outcomes; early positive fluid balance is significantly associated with hypertension, peripheral edema, pulmonary edema, respiratory failure, onset of ARDS, increased cardiac demand, prolonged mechanical ventilation, longer ICU stay, and an increased risk of death [119,120,121,122,123]. The Fluids and Catheters Treatment Trial (FACTT), conducted in 2006 by Wiedemann and co-authors, further supports these findings. It demonstrates that a restrictive fluid strategy, leading to a reduced fluid balance in patients with ARDS and guided by hemodynamic and clinical variables, is linked to better outcomes, improved oxygenation, and a shorter duration of mechanical ventilation [124]. Similarly, fluid accumulation can have devastating effects in neurocritical care patients by exacerbating cerebral edema in those with BBB disruption, which is associated with increased ICU mortality and worse outcomes [5,125]. Indeed, a prospective, multicenter, comparative effectiveness study of two observational cohorts (CENTER-TBI and the OzENTER-TBI Collaboration Groups), including 2125 adult patients with TBI, demonstrated that a mean positive daily fluid balance was associated with higher ICU mortality and worse functional outcomes. Additionally, higher mean daily fluid input was also linked to higher ICU mortality [5]. Moreover, a retrospective study of 351 patients with moderate and severe TBI highlighted that both low and high fluid balance were associated with poor short-term outcomes. Fluid balance in the upper tertile was potentially an independent predictor of poor 30-day outcomes, mainly linked to acute kidney injury and refractory intracranial hypertension [126].

Considering the above, fluid management should aim at maintaining a stable hemodynamic status, avoiding both hypovolemia and hypervolemia, and be guided by sufficient invasive and noninvasive monitoring, as suggested by current guidelines for the management of TBI patients, in order to prevent secondary complications associated with fluid imbalance [127,128].

#### 3.1.3. CPP and ICP

ICP refers to the pressure within the cranial cavity, which is determined by the combined volumes of brain tissue, vascular compartments, and CSF. According to the Monro–Kellie hypothesis, any increase in the volume of one of these components must be balanced by a decrease in one or both of the others to maintain stable ICP, as failure of this compensatory mechanism can lead to harmful consequences [129]. Experimental studies have shown that intracranial hypertension is associated with the development of focal ischemia in critical regions of the brainstem due to transtentorial pressure gradients. Additionally, it can lead to compression of the bridging veins, resulting in elevated cerebral venous pressure, a critical reduction in CPP, and subsequent cerebral hypoperfusion [130,131].

To improve in-hospital and 2-week post-injury mortality, the current BTF guidelines recommend an ICP threshold of 22 mmHg, while the suggested CPP range has been refined to 60–70 mmHg [111]. However, the ESICM consensus in October 2016 did not provide recommendations on whether an isolated ICP threshold of 20–22 mmHg, without considering other variables, should be used as an indication to initiate osmotherapy. Nonetheless, they propose a threshold higher than 25 mmHg, independent of other variables, as a trigger for treating intracranial hypertension with osmotherapy [18].

Determining safe ICP and CPP levels remains an area of ongoing research, particularly in relation to the patient’s cerebral autoregulation capacity. Current evidence supports maintaining ICP below 22 mmHg and cerebral CPP between 60 and 70 mmHg. Deviations beyond these thresholds—such as ICP exceeding 25 mmHg or CPP falling below 50 mmHg or above 70 mmHg—are associated with an increased risk of secondary brain injury [111,113,132]. However, accumulating evidence over recent decades supports a personalized therapeutic approach based on optimal cerebral perfusion pressure (CPPopt), which involves identifying changes in ICP in relation to arterial blood pressure fluctuations. This relationship can be quantified using the pressure reactivity index (PRx) [133], a variable that indicates cerebral autoregulation [134]. Under normal conditions, a rise in MAP leads to a decrease in ICP, indicated by a negative PRx, which suggests intact cerebral autoregulation. In contrast, a positive PRx is seen when autoregulation is impaired, where an increase in MAP results in an elevation of ICP [106]. Proposed threshold values for PRx relevant to prognosis are +0.25 and +0.05 for mortality and poor outcome, respectively [135]. The collection and plotting of PRx and CPP values over time typically produce a U-shaped curve, where the lowest point corresponds to the CPPopt [133]. Previous studies have shown a strong link between CPPopt and patient outcomes, indicating that maintaining CPP close to the dynamically adjusted CPPopt is associated with improved six-month outcomes [136,137,138,139]. Deviations from CPPopt, particularly values below it, have been associated with higher mortality in TBI patients, while values significantly above CPPopt are linked to increased risk of disability [140]. Proposed threshold values for PRx relevant to prognosis are +0.25 and +0.05 for mortality and poor outcome, respectively [135]. A phase II prospective randomized controlled trial (RCT), the COGiTATE study (CPPopt Guided Therapy: Assessment of Target Effectiveness), compared CPPopt-guided therapy with standard care as recommended by the Brain Trauma Foundation (CPP thresholds of 60–70 mmHg). While the CPPopt group showed lower mortality and better recovery, these differences did not reach statistical significance—likely due to the fact that COGiTATE was designed as a feasibility trial [141]. Nonetheless, a recent study analyzing data from 809 neurocritically ill patients with TBI who underwent invasive ICP monitoring found that patients with CPP values below individualized thresholds had worse outcomes compared to those with values above these thresholds [142]. These findings are further supported by a multicenter study involving 432 TBI patients, which demonstrated a correlation between CPPopt and maximized partial pressure of brain tissue oxygen (PbtO_2_), emphasizing CPPopt as a key physiological variable in therapeutic management. However, maintaining ICP within predefined targets (<20 mmHg) was strongly associated with prognosis, suggesting that ICP control should be the primary therapeutic goal, followed by the optimization of PbtO_2_ and CPPopt [143]. Nonetheless, if CPPopt is found to be outside the typical range of 60–70 mmHg, this may indicate that the patient could benefit from a blood pressure challenge [144]. Phase III trials and expert consensus are needed to establish the therapeutic value of CPPopt-guided management. Current guidance recommends an ICP threshold of 22 mmHg and a CPP range of 60–70 mmHg [111], personalized according to CPPopt when available. CPP targets above 70 mmHg should be approached with caution and carefully assessed [91], as CPP values exceeding 70 mmHg have been linked to pulmonary complications such as ARDS and poorer outcomes [145].

### 3.2. Monitoring

#### 3.2.1. Hemodynamic Monitoring

Fluid responsiveness refers to the left ventricle’s ability to enhance stroke volume (SV) following fluid administration [146]. The passive leg raise (PLR) test and the end-expiratory occlusion test have been established as dynamic and effective screening tools for assessing volume responsiveness [147,148]. However, their use in brain-injured patients remains controversial due to concerns about potential increases in cerebral blood volume, which could worsen intracranial hypertension and disrupt cerebrovascular reactivity [149,150,151]. Indeed, a recent prospective, observational, single-center study in critically ill patients with stable acute brain injury (ABI) receiving invasive ICP monitoring found that the PLR test led to ICP elevation, mostly remaining within safe levels. Nevertheless, persistent cerebral autoregulation disturbances lasting up to 10 min were observed, suggesting that PLR should be avoided in ABI patients, even in those with stable ICP [149]. As an alternative to PLR, potential targets for guiding personalized fluid resuscitation may include estimation of capillary refill time, repeated lactate measurements, echocardiography, ScvO_2_, a “mini fluid challenge” and invasive hemodynamic monitoring [152,153].

In the past, CVP was recommended as a tool to assess fluid responsiveness and guide fluid administration [154]. However, subsequent research has established that static variables such as CVP do not sufficiently estimate fluid responsiveness in both mechanically ventilated and spontaneously breathing patients [155,156,157,158,159]. For these reasons, a task force of the ESICM, approximately 10 years ago, recommended the use of advanced hemodynamic monitoring in critically ill patients, including those with ABI, to evaluate hemodynamic status and assess volume responsiveness in complex medical scenarios [160].

The transpulmonary thermodilution (TPTD) method is an advanced hemodynamic monitoring technique that, despite being invasive, requires only the insertion of a central venous catheter and an arterial thermistor catheter, allowing measurements of CO, intrathoracic blood volume, and extravascular lung water (EVLW) [161]. Indeed, beyond CO measurements, TPTD allows the determination of various hemodynamic parameters, such as pulse pressure and stroke volume variation to guide fluid management, EVLW to assess lung permeability and pulmonary edema, ejection fraction to evaluate left ventricular function, and global end-diastolic volume to reflect cardiac preload [162,163]. In a survey by the ESICM, Messina et al. (2022) [164] highlight the heterogeneity of clinical approaches to hemodynamic management in brain-injured patients across various centers, showing that advanced hemodynamic monitoring is primarily used in those with more severe diseases. However, they also suggest potential beneficial effects in hemodynamically stable patients [164]. Nonetheless, data regarding the use of TPTD in patients with TBI are scarce. In addition, one might argue that, given the improved outcomes observed in patients with other causes of brain injury—such as SAH—where TPTD-directed fluid management has been associated with a decreased incidence of delayed cerebral ischemia and more stable systemic hemodynamics, patients with TBI may also benefit from such an approach [165,166,167]. Therefore, conducting research in this area is essential to determine its potential benefits for TBI patients.

#### 3.2.2. Neuromonitoring

Multimodal monitoring in the management of TBI refers to the use of multiple non-invasive and invasive methods, or a combination of both, to individualize a patient’s therapeutic strategy and prevent secondary brain injury. This includes clinical examination, radiologic modalities, monitoring of intracranial and perfusion pressures, brain tissue oxygenation and metabolism, cerebral autoregulation, and electrophysiology [168,169]. ICP monitoring is the cornerstone of the therapeutic strategy for patients with severe ABI, including TBI, fatal stroke, and intracerebral bleeding. ICP acts as an indicator of expanding intracerebral mass lesions and plays a role in the early diagnosis of imminent herniation. Moreover, ICP monitoring is a key diagnostic tool for detecting intracranial hypertension and enables the calculation of CPP (as the difference between MAP and ICP), which is the key regulator of oxygen and substrate delivery to the brain [170,171,172]. In recent years, it has been convincingly demonstrated that ICP monitoring in patients with severe TBI is associated with better outcomes compared to those without it, leading to the recommendation of ICP monitoring and CPP calculation to prevent secondary brain injury [173,174]. Indeed, research indicates that patients with severe TBI who underwent ICP monitoring received more intensive management and had better outcomes, as evidenced by significantly lower 6-month mortality compared to those who were not monitored [175].

However, normal brain tissue oxygenation cannot be guaranteed even if MAP, ICP, and consequently CPP levels are within the targeted thresholds [176]. Therefore, multimodal neuromonitoring, including additional variables such as PbtO_2_, has been suggested [177,178]. PbtO_2_ is an invasive monitoring tool that serves as a marker of regional brain oxygenation [179]. In patients with TBI, targeted PbtO_2_ values are generally considered to be above 15 mmHg [180] and even above 20 mmHg [181], as values below the 15–20 mmHg range are associated with poor outcomes [111,182]. The rationale for combining PbtO_2_ and ICP monitoring is to facilitate the early diagnosis of reduced cerebral perfusion and minimize the duration of cerebral hypoxic episodes, thereby limiting secondary brain injury [179,183,184]. Indeed, the BOOST-II trial highlighted that multimodal monitoring of ICP and brain tissue oxygenation in patients with TBI helps to eliminate brain tissue hypoxia and is associated with a potential reduction in mortality and improved outcomes compared to ICP-guided treatment alone. The study also proposed four different management approaches based on various combinations of ICP and PbtO_2_ values (neither deviated, both deviated, or one deviated), which have been adopted in the SIBICC (Seattle International Severe Traumatic Brain Injury Consensus Conference) recommendations for TBI management [127,185,186].

PbtO_2_-guided fluid resuscitation, in conjunction with CPP and ICP monitoring as well as assessment of cerebral autoregulation, may help optimize CPP targets. The American College of Surgeons, in their recent 2024 guidelines for TBI management, even recommend transfusing one unit of red blood cells if PbtO_2_ persists below 20 mmHg despite CPP optimization [187]. A similar approach has been applied in patients with SAH, where episodes of brain tissue hypoxia, defined as PbtO_2_ < 20 mmHg for more than 10 min, were managed with vasopressors to achieve CPP ≥ 70 mmHg, fluid resuscitation to preserve euvolemia, and red blood cell transfusion [188]. However, data regarding PbtO_2_-guided hemodynamic management in patients with TBI are scarce. In a small prospective study of 11 patients with ABI, Johnston et al. (2005) demonstrated that increasing MAP from 70 to 90 mmHg with norepinephrine improved CBF, increased PbtO_2_, and decreased oxygen extraction fraction [189]. Recently, Kunapaisal et al. (2024), in a retrospective analysis of prospectively collected data from 93 patients with severe TBI, reported that MAP elevations led to increases in CPP but only modestly affected PbtO_2_, resulting in four distinct PbtO_2_ response patterns [190]. The ongoing Brain Oxygen Optimization in Severe Traumatic Brain Injury phase III trial (BOOST-3; NCT03754114) is a multicenter, randomized, blinded-endpoint comparative effectiveness study enrolling 1094 patients with severe TBI. It is investigating neurological outcomes at 180 days when therapeutic strategies using vasoactive agents or fluid administration are guided by ICP alone (with the treating team blinded to PbtO_2_ values) versus by both ICP and PbtO_2_ [190,191].

Cerebral microdialysis (MD) is an invasive neuromonitoring method used to assess cerebral metabolism in neurocritically ill patients with brain injury, including those with TBI [192,193], with the aim of preventing secondary brain injury [193,194]. In recent years, cerebral MD has evolved into a significant neuromonitoring tool alongside ICP monitoring and PbtO_2_ in specialized neuro-ICUs to optimize therapeutic strategies. Studies have demonstrated that MD may contribute to optimizing CPP and oxygen therapy, guiding glucose control and nutrition, and informing red blood cell transfusions. Moreover, it serves as an advanced research tool to investigate various pathophysiological mechanisms following TBI, including brain biochemistry and energy disturbances, neuroinflammation, and BBB alterations [195].

## 4. Fluid Administration in TBI

### 4.1. Types of Fluids

#### 4.1.1. Colloids vs. Crystalloids

Maintaining normovolemia—defined as a neutral or net zero fluid balance, according to the CENTER-TBI and OzENTER-TBI collaboration groups [5]—through non-invasive and invasive hemodynamic monitoring is critical in patients with TBI [18,113]. However, data on achieving and maintaining a normovolemic hemodynamic status in TBI patients remain limited [113].

Intravenous fluid resuscitation can be performed using either crystalloids or colloids. Crystalloids contain electrolytes dissolved in water and freely distribute between blood vessels and the interstitial space. Colloids contain large molecular particles that generally cannot cross capillary membranes under normal conditions [196]. Indeed, due to their molecular weight, colloids remain in the intravascular compartment longer, leading to greater volume expansion, an increase in colloid osmotic pressure, and consequently, a ‘volume-sparing’ effect [197,198].

#### 4.1.2. Albumin vs. Crystalloids

According to the ESICM consensus (2018), crystalloids should be the first-line fluid choice for neurocritically ill patients with hypotension [18]. Nevertheless, it has also been suggested that normovolemia should be achieved through albumin administration and red blood cell transfusions [199]. However, the administration of albumin in patients with TBI remains a subject of controversy given the contradictory results of clinical studies, with some demonstrating beneficial effects in cerebral edema and hemodynamic stabilization [113,200] and others highlighting increased mortality, particularly in patients with severe TBI [30]. In addition to the increased mortality of TBI patients receiving albumin, the post hoc analysis of the SAFE study with long-term follow-up revealed that patients in the albumin group (receiving 4% albumin) had higher two-year mortality and poorer neurological outcomes compared to those receiving crystalloids. Nonetheless, the evidence is considered uncertain [30]. The elevated mortality in the 4% albumin group was potentially associated with an increase in cerebral edema and elevated ICP. It was hypothesized that the underlying pathophysiological mechanism involved enhanced translocation of albumin from the intravascular space into the interstitial brain tissue due to alterations in the BBB [30,201]. On the other side, previous research on patients with SAH demonstrated better neurological prognosis in those who received high-dose albumin compared to crystalloids [202,203]. In addition, a phase I dose-escalation study evaluating the safety of albumin therapy in patients with acute ischemic stroke highlighted that treatment with high-dose albumin (25%) resulted in better neurological outcomes at three months, emphasizing its potential neuroprotective properties [204]. These data are further supported by experimental animal models of cerebral ischemia, showing the neuroprotective abilities of high-dose albumin (20 to 25%) on neurological outcomes, infarction size, and cerebral edema [205,206,207,208]. Nevertheless, a multicenter, randomized, double-blind, parallel-group, placebo-controlled trial failed to demonstrate beneficial effects of using 25% albumin in patients with acute ischemic stroke [209].

Based on these controversial findings and considering the higher cost of albumin compared to crystalloids, its potential impact on healthcare resource availability, its limited supply in certain situations, and the fact that it is a blood product, the ESICM recently recommended against the use of albumin in critically ill patients with TBI in its clinical practice guidelines on resuscitation fluids for adult critically ill patients [210].

#### 4.1.3. Balanced Crystalloids vs. Isotonic Saline

Administration of isotonic saline is one of the most common interventions in the ICU [211]. Normal saline contains supraphysiologic chloride concentrations (154 mmol/L), which have been associated with the development of hyperchloremia and hyperchloremic metabolic acidosis when large volumes are administered [212,213]. Moreover, in critically ill patients, the administration of chloride-liberal fluids has been identified as a risk factor for acute kidney injury, due to reductions in renal perfusion, immunosuppression, and increased mortality [214,215]. These findings have led to recommendations favoring the use of balanced crystalloids over normal saline in critically ill patients, with exceptions including neurocritical patients, such as those with TBI, and patients with alkalosis and hypochloremia due to profound vomiting, who may benefit from normal saline [210,216,217]. However, in patients with TBI, fluid selection must balance the risk of hyperchloremic metabolic acidosis and acute kidney injury associated with normal saline against the potential for exacerbating cerebral edema due to the relative hypotonicity of most balanced solutions [218,219,220,221]. Notably, the relative hypotonicity of balanced crystalloids—characterized by lower concentrations of sodium and chloride, which are partially replaced by organic anions and other cations—may make patients with TBI more vulnerable to worsening cerebral edema [94,105,212]. Indeed, a secondary analysis of the PROMMTT trial (PRospective Observational Multicenter Major Trauma Transfusion) [222] highlighted that patients with TBI who received Ringer’s lactate in the prehospital setting had higher mortality compared to those who received isotonic saline [223]. These findings align with those of a subgroup analysis of patients with TBI in the SMART trial (Isotonic Solutions and Major Adverse Renal Events Trial) [224], which demonstrated that patients who received balanced crystalloids had an increased likelihood of mortality and a worse discharge disposition compared to those in the isotonic saline group [218]. Moreover, a recent systematic review and meta-analysis including 1896 patients with TBI found a 60% relative increase in the risk of death in the balanced crystalloids group compared to the saline group [225]. Recently, the clinical practice guidelines on fluid therapy from the ESICM noted that normal saline is preferred over balanced crystalloids for fluid resuscitation in patients with TBI, and advised against the use of Ringer’s lactate (or acetate) in these patients [210]. Notably, although Plasma-Lyte is closer to plasma in terms of pH and electrolyte composition and has a more favorable metabolic profile than Ringer’s lactate in critically ill patients, both solutions are relatively hypotonic compared to 0.9% saline, which may increase the risk of cerebral edema in patients with TBI [218,226,227].

#### 4.1.4. Hypertonic Saline

Although fluid resuscitation is one of the most frequent therapeutic interventions to restore and maintain tissue perfusion in critically ill patients, volume overload is associated with increased mortality [228,229], prolonged ICU stays [230], fewer ventilator-free days [231], respiratory complications [232,233], major adverse kidney events [234], coagulopathy [235], hypothermia [236], and acidosis [237], especially if large amounts of non-balanced crystalloids as isotonic saline are infused rapidly. Hypertonic saline is a widely available and low-cost therapy, which has been used in various concentrations for fluid resuscitation in critically ill patients with hypovolemic shock due to its ability to rapidly expand intravascular volume with a lower overall infused volume [238,239]. Indeed, hypertonic fluids create an osmotic gradient within the vascular space, drawing fluid in from the interstitial and intracellular compartments [240]. Moreover, although still a subject of controversy, the effectiveness of hyperosmolar solutions in rapidly reducing ICP is well established [241,242,243,244,245]. Furthermore, it has been shown that hypertonic saline can improve systemic hemodynamics by increasing CO and reducing peripheral vascular resistance through modifications of myocardial contractility, capillary systemic vasculature, mitigation of myocyte edema, and enhancement of cardiac calcium uptake [246,247,248]. Finally, research highlights the anti-inflammatory and immunomodulatory properties of hypertonic saline, which may contribute to its beneficial effects [249]. However, since the use of hypertonic solutions is not associated with benefits in patients with TBI compared to isotonic saline, their use as resuscitation fluid is not recommended [18,250].

## 5. Transfusion Strategies in Patients with TBI

Anemia is highly prevalent among ICU patients and is associated with detrimental outcomes, such as myocardial injury [251], prolonged mechanical ventilation [252], extended hospital stays, and increased mortality [253,254]. However, blood product transfusions are linked to adverse events, including volume overload, lung injury, and secondary infections [255,256,257]. Transfusion thresholds and hemoglobin targets in the absence of life-threatening hemorrhage remain uncertain [258,259]. In recent years, it has been highlighted that restrictive transfusion strategies are as safe as, or safer than, more liberal approaches in most patient groups [257,260,261]. According to recent guidelines from the ESICM, a restrictive transfusion strategy is recommended over a liberal transfusion strategy (hemoglobin 7 g/dL vs. 9 g/dL, respectively) in critically ill patients in the general ICU, with or without ARDS [37]. Notably, the optimal transfusion strategy remains uncertain for specific patient populations, such as those with ABI [38,39]. Indeed, due to the high cerebral metabolic demands, patients with ABI may benefit from a liberal transfusion strategy, potentially improving oxygen delivery capacity and reducing the risk of brain tissue hypoxia and secondary brain injury, particularly in the setting of impaired cerebral autoregulation [257,261,262].

Severe anemia often coexists with moderate to severe TBI, occurring in approximately half of patients within the first 48 h after the traumatic event, and significantly influences patient outcomes [263,264]. The pathophysiology of anemia in patients with TBI is complex and involves multiple mechanisms, including traumatic and perioperative acute blood loss, hemodilution from aggressive fluid resuscitation, impaired erythropoiesis, phlebotomy losses, hemolysis, reduced red blood cell proliferation due to inflammatory responses, and iron and vitamin deficiencies [262,265,266,267,268].

Experimental research highlights the detrimental effects of anemia in animal models of TBI, demonstrating reductions in PbtO_2_ and oxygen extraction, intracranial hypertension, anemia-associated cellular injury, and cerebral edema, which contribute to secondary brain damage, hypoxic cerebral injury, and poor prognosis [264,269]. These findings are consistent with results from clinical studies. A recent secondary analysis of the CENTER-TBI study showed that increases in hemoglobin were associated with better neurological outcomes and reduced mortality, whereas hemoglobin levels below 7.5 g/dL were linked to unfavorable neurological outcomes and increased mortality [270]. The TRAIN study enrolled 850 patients with ABI (including TBI) to compare liberal (transfusion threshold 9 g/dL) and restrictive (transfusion threshold 7 g/dl) transfusion strategies. Patients in the liberal group had a lower risk of poorer neurological outcomes at 6 months compared to those in the restrictive group [259]. These findings are consistent with those of a previous single-center study that compared outcomes between restrictive and liberal transfusion strategies using hemoglobin thresholds of 7 g/dL and 9 g/dL, respectively, in patients with moderate or severe TBI. The study reported improved mortality rates and better neurological outcomes at 6 months in the liberal transfusion group compared to patients who received fewer transfusions (62% vs. 44%) [271]. However, the EPO Severe TBI trial randomized patients with closed head injuries to receive either a liberal transfusion strategy with a transfusion threshold of 10 g/dL or a restrictive approach with a threshold of 7 g/dL. The study found no improvement in neurological outcomes at 6 months for the liberal strategy compared to the restrictive approach, while patients in the liberal group had a higher incidence of adverse events [272]. Moreover, a recently published study in patients with moderate to severe TBI (HEMOTION study) failed to demonstrate a beneficial effect of a liberal (transfusion threshold 10 g/dL) compared to a restrictive (transfusion threshold 7 g/dL) transfusion strategy on mortality and neurological outcomes at six months, although the liberal strategy was associated with better scores on some scales evaluating functional independence and quality of life [273]. However, caution is needed when interpreting the findings of the aforementioned studies, as they employed different transfusion thresholds (10 g/dL vs. 9 g/dL; 7.5 g/dL vs. 7 g/dL), involved different patient populations (TBI vs. ABI in the TRAIN trial), and used varying definitions of poor neurological outcome. Additionally, the inclusion of patients receiving erythropoietin in the study by Robertson et al. (2014) represents a further methodological difference compared to the TRAIN and HEMOTION trials [259,270,271,272,273]. The hemoglobin thresholds applied in clinical studies of transfusion strategies in patients with TBI are summarized in Table 1. While awaiting the evaluation of the results from the TRAIN and HEMOTION trials, it is suggested that the transfusion strategy in patients with severe TBI be tailored to individual systemic and cerebral physiological parameters [127].

## 6. Conclusions

Maintenance of CPP and prevention of secondary brain injury are critical goals in the management of patients with TBI, frequently necessitating fluid resuscitation and vasoactive support. A consensus or standardized approach to fluid resuscitation management in patients with TBI does not exist. Notably, the goal of resuscitation should be to maintain normotensive blood pressure levels and euvolemia, as both hypotension and hypertension, as well as hypovolemia and hypervolemia, can have detrimental effects. Careful administration of crystalloids guided by hemodynamic and multimodal neuromonitoring is the therapy of choice, while hypertonic solutions could be considered in patients with increased ICP. Balanced crystalloids are not suggested, as their use could theoretically be associated with the development or worsening of cerebral edema. Given the uncertainty of existing data, the transfusion approach should be adjusted to individual systemic and cerebral physiological parameters.

## 7. Key Take-Home Points

1. Key elements of TBI management are mainly ICP monitoring and maintenance of CPP to avoid or minimize secondary brain injury, limit cerebral edema, and reduce primary neuronal damage.
2. Current guidelines recommend an ICP threshold of <22 mmHg and a CPP of 60–70 mmHg, personalized according to CPPopt when available.
3. The goal of fluid resuscitation is to maintain normotensive blood pressure and euvolemia.
4. Both hypotension and hypertension, as well as hypovolemia and hypervolemia, can have harmful effects.
5. Crystalloid administration based on hemodynamic and multimodal neuromonitoring is the therapy of choice, while hypertonic solutions may be considered in patients with intracranial hypertension.
6. Balanced crystalloids are generally not recommended, as their use could theoretically contribute to the development or worsening of cerebral edema.
7. Transfusion strategies in patients with severe TBI should be tailored to individual systemic and cerebral physiological parameters.

## Figures and Tables

**Table 1:** Overview of hemoglobin thresholds used in clinical studies investigating transfusion strategies in patients with traumatic brain injury.

Study	Liberal	Restrictive
Guglielmi A. et al. (2024) [270]	7.5–9.5 g/dL	<7.5 g/dL
Taccone F.S. et al. (2024) [259]	9 g/dL	7 g/dL
Gobatto A.L.N. et al. (2019) [271]	9 g/dL	7 g/dL
Robertson C.S. et al. (2014) [272]	10 g/dL	7 g/dL
Turgeon A.F. et al. (2024) [273]	10 g/dL	7 g/dL

## Data Availability

No new data were created or analyzed in this study.
